# Polyvinyl-Pyrrolidone-Coated Silver Nanoparticles—The Colloidal, Chemical, and Biological Consequences of Steric Stabilization under Biorelevant Conditions

**DOI:** 10.3390/ijms22168673

**Published:** 2021-08-12

**Authors:** Andrea Rónavári, Péter Bélteky, Eszter Boka, Dalma Zakupszky, Nóra Igaz, Bettina Szerencsés, Ilona Pfeiffer, Zoltán Kónya, Mónika Kiricsi

**Affiliations:** 1 Department of Applied and Environmental Chemistry, Faculty of Science and Informatics, University of Szeged, H-6720 Szeged, Hungary; ronavari@chem.u-szeged.hu (A.R.); peti0225@gmail.com (P.B.); bokaeszti@gmail.com (E.B.); z.dalma.ballet@gmail.com (D.Z.); 2 Department of Biochemistry and Molecular Biology, Faculty of Science and Informatics, University of Szeged, H-6726 Szeged, Hungary; noraigaz@gmail.com (N.I.); kiricsim@gmail.com (M.K.); 3 Department of Microbiology, Faculty of Science and Informatics, University of Szeged, H-6726 Szeged, Hungary; betti414@gmail.com (B.S.); pfeiffer@bio.u-szeged.hu (I.P.); 4 MTA-SZTE Reaction Kinetics and Surface Chemistry Research Group, H-6720 Szeged, Hungary

**Keywords:** chemical stability, steric stabilization, aggregation behavior, antimicrobial activity, cytotoxicity, anticancer activity

## Abstract

(1) Background: Several properties of silver nanoparticles (AgNPs), such as cytotoxic, anticancer, and antimicrobial activities, have been subjects of intense research; however, important aspects such as nanoparticle aggregation are generally neglected, although a decline in colloidal stability leads to a loss of the desired biological activities. Colloidal stability is affected by pH, ionic strength, or a plethora of biomolecules that interact with AgNPs under biorelevant conditions. (2) Methods: As only a few studies have focused on the relationship between aggregation behavior and the biological properties of AgNPs, here, we have systematically evaluated this issue by completing a thorough analysis of sterically (via polyvinyl-pyrrolidone (PVP)) stabilized AgNPs that were subjected to different circumstances. We assessed ultraviolet–visible light absorption, dynamic light scattering, zeta potential measurements, in vitro cell viability, and microdilution assays to screen both colloidal stability as well as bioactivity. (3) Results: The results revealed that although PVP provided outstanding biorelevant colloidal stability, the chemical stability of AgNPs could not be maintained completely with this capping material. (4) Conclusion: These unexpected findings led to the realization that stabilizing materials have more profound importance in association with biorelevant applications of nanomaterials than just being simple colloidal stabilizers.

## 1. Introduction

Due to the numerous favorable features of nanomaterials, their application in various fields, including medical, food, consumer, health care, and industrial purposes, is constantly increasing. However, characterization of their chemical and biological behavior is fundamental to ensure safe and predictable application, especially upon encountering living organisms. In recent years, the design and fabrication of next-generation silver-based nanomaterials have been subjects of intense research in the field of material sciences [1,2]. Silver nanoparticles (AgNPs) are considered to be one of the most heavily investigated, most frequently synthesized and commercialized nanomaterials owing to their optical, electrical, and thermal features as well as catalytic activities [3,4].

In the biomedical field, the excellent properties of nanosilver are especially exploited since AgNPs are known to exhibit outstanding antimicrobial, anti-inflammatory, anticancer, and antiangiogenic activities. These unique chemical, physical, and biological properties of AgNPs are massively influenced by a number of factors such as nanoparticle size and morphology or by surface coating, which are generally determined at nanoparticle synthesis [5,6,7]. Therefore, proper selection of the synthesis method is crucial for achieving the desired particle properties for specific applications [8,9]. In the last decade, a plethora of procedures have been developed for AgNP production; however, the most common approach, which is also highly effective and easy to control, is the chemical reduction of a silver salt using various reducing and capping agents [10].

Employment of nanoparticles for any function requires nanoparticles of reliable long-term stability, with controlled and well-defined properties. In fact, colloidal stability and aggregation propensity are issues that should be more profoundly regarded. Aggregation might cause a substantial decrease in the effective surface area of nanomaterials, which can lead to the reduction or complete loss of the advantageous nano-properties [11]. As the synthesis method might also govern the lasting stability of silver nanoparticle suspensions, numerous chemical and biological approaches have been adopted and optimized (reaction circumstances, reducing and stabilizing agents) to improve the parameters influencing the stability of silver nanoparticles [12,13]. Different types of capping and stabilizing agents, such as surfactants (e.g., cetrimonium bromide (CTAB)), polymers (e.g., polyvinyl pyrrolidone (PVP)), and green materials (such as extracts of plant parts, e.g., tea leaves or coffee beans) have been utilized for the surface modification of AgNPs to prevent aggregation [14,15,16]. Various stabilization mechanisms such as electrostatic, steric, and electrosteric stabilization were engaged upon synthesis, yielding nanoparticles with different physical, chemical, and biological characteristics and colloidal stability [17]. The colloidal stability of nanoparticles designed for therapeutic utilization is absolutely essential since nanoparticle aggregation in living systems, under biorelevant conditions, can not only directly determine the biological (such as antimicrobial, cytotoxic, or drug delivery) activity of the particles, but it can also have life-threatening consequences [15]. It is therefore intriguing that although plenty of studies have reported how the various reducing and stabilizing entities affect the size, size distribution, and shape as well as the biological activity of AgNPs, nevertheless, the scientific literature is largely lacking in data on the aggregation behavior of nanoparticles, especially the effects that particle aggregation exerts on the bioactive features of the produced nanomaterials.

In our recently published papers, we described environmentally benign synthesis approaches to AgNP formation, and we also showed that the various green materials used for stabilization and reduction of metal ions have a defining role in determining the colloidal stability and biological activity of the obtained green nanoparticles [8,18]. The effect of particle size on the aggregation behavior of different AgNP suspensions and the modulation of antimicrobial activity and toxicity as the function of nanoparticle aggregation were also investigated. Thereafter, we systematically analyzed and compared the aggregation behavior of electrosterically and electrostatically stabilized AgNPs in different biological systems using an experimental procedure consisting of a series of chemical and in vitro biological measurements [19]. We demonstrated that the electrostatically stabilized AgNPs completely lost their biological activity due to aggregation, while the electrosterically stabilized AgNPs were less prone to aggregate under biorelevant experimental conditions. Through these experiments, we highlighted the need for careful selection of the proper nanomaterial synthesis method to achieve the desired particle properties and raised awareness for a compulsory comprehensive chemical and biological screening procedure to assess the stability and aggregation propensity of nanoparticles to estimate their fate and behavior under biorelevant conditions prior to their application. However, sterically stabilized silver nanoparticles have never been involved in a systematic evaluation of the aggregation–biological activity relationship; therefore, the present paper aims to fill that void in order to provide a complete view on the possible nanoparticle stabilization types regarding this issue. For this purpose, the most common steric stabilizer, the natural biopolymer PVP, was used to produce silver nanoparticles. The obtained particles were examined thoroughly under biorelevant conditions to delineate the effects of varying pH and differing concentrations of sodium chloride, glucose, glutamine, and fetal bovine serum (FBS) on particle stability. Moreover, the aggregation behavior of these PVP-stabilized particles was measured in a time-dependent manner to evaluate the nanoparticle aggregation grade and its impact on cytotoxicity and antimicrobial activity in the function of time. This experimental setup provided further evidence on the profound significance of the degree of silver nanoparticle aggregation in modulating biological activity against cancerous and non-cancerous human cells as well as various microbes and how this feature could be affected, in particular, by steric stabilization.

## 2. Results and Discussion

### 2.1. Nanoparticle Morphology and Crystallinity

**Figure 1:**
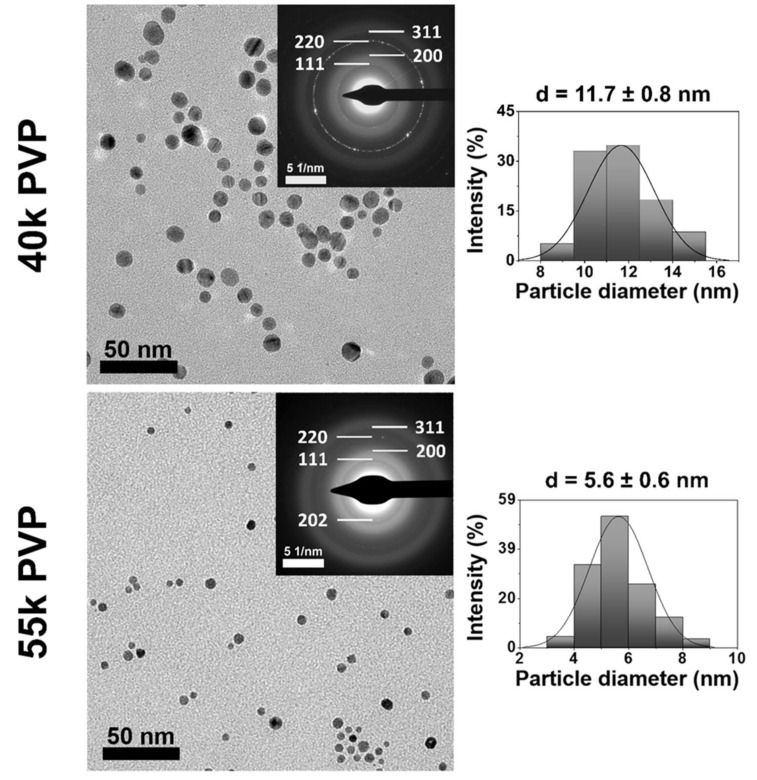
Transmission electron microscopic (TEM) images, electron diffraction (ED) patterns, and size distribution histograms of silver nanoparticles stabilized by 40k (top) and 55k (bottom) molecular weight PVP.

The morphological properties of the synthesized AgNPs are presented in Figure 1 with the corresponding TEM images, electron diffraction patterns, and their respective particle size distribution histograms. Both PVP-utilizing reactions resulted in spherical nanoparticles. The smaller molecular weight (MW) PVP-stabilized system yielded larger particles, around 12 nm, while the larger molecular weight system generated smaller particles of about 6 nm diameter in accordance with the relevant literature. In their contribution, Madkour et al. proposed that during nanoparticle synthesis, one end of the stabilizing polymers may attach to the forming particles, while the rest of the chain forms a loose adsorbate layer around them [20]. The degree of coverage around the particles increases with polymer molecular weight, thus inhibiting further Ag^+^ reduction on the surface to a higher degree, resulting in smaller particles compared to systems utilizing smaller MW stabilizers, where the particles have a larger free surface, rendering particle growth possible. The sample prepared with 40k PVP demonstrated prominent Debye-Scherrer rings, corresponding to 2.35 and 1.44 Å lattice distances, or Miller indices of (111) and (220), respectively; to a smaller degree, rings corresponding to (220) and (311) were detected, verifying the chemical composition of the particles [8,18,21,22]. AgNPs synthesized by 50k PVP also demonstrated these rings, although in a much more diffuse manner, due to their smaller particle size. Furthermore, an additional ring appeared, which, according to the Inorganic Crystal Structure Database (ICSD), corresponds to the (202) crystal facet of silver oxide (2.73 Å), demonstrating the somewhat reduced chemical uniformity of silver nanoparticles stabilized through 50k PVP [23].

### 2.2. Chemical Stability Analysis

**Figure 2:**
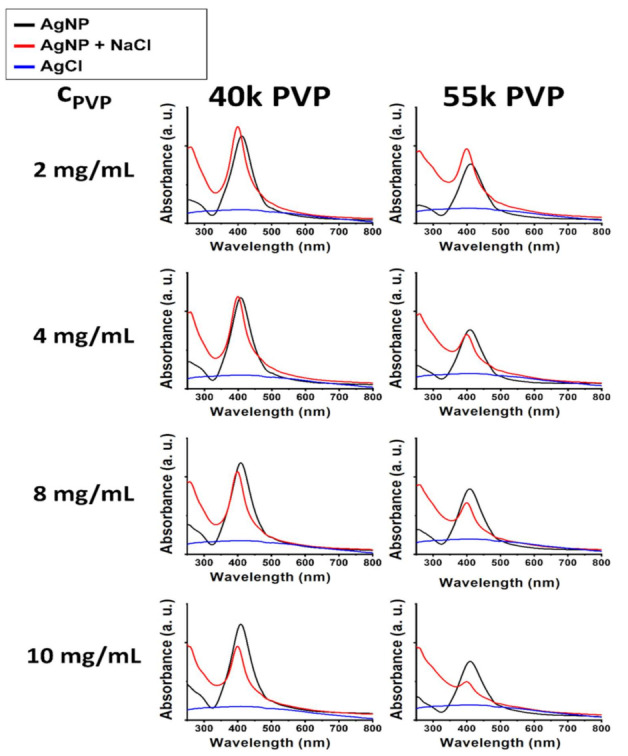
UV–Vis spectra demonstrating the chemical degradation of 40k (left panels) and 55k (right panels) molecular weight PVP-stabilized silver nanoparticles through AgCl precipitation induced by incubation with 100 mM NaCl for 20 h under various PVP concentrations (indicated on the left side of the panels).

According to the literature and our previous results, the characteristic surface plasmon peaks of silver nanoparticles can undergo various changes due to interfering agents in the liquid media [16,18,24]. Their λ_max_ may redshift due to surface interactions, and their absorbance intensities can decrease with aggregation [25,26]. However, the results in Figure 2, describing the effect of NaCl on silver nanoparticles stabilized by various concentrations of PVP, cannot be explained by the above-mentioned phenomena. Each spectrum, corresponding to AgNP samples mixed with 100 mM NaCl, demonstrated slight SPR blue shifts and absorbance increases at the local minima around 325 nm compared to the untreated AgNP samples. Blue shifts may occur due to a decrease in nanoparticle diameter, while the intensity increase of the absorbance valley can be explained by the formation of AgCl precipitation [24,27].

The UV–Vis results implied that all measured samples are prone to decomposition upon surface contact with Cl^−^ ions through AgCl formation, although to varying degrees. As these experiments were aimed at aiding in the selection of the most proper AgNP@PVP system, to be examined in the subsequent part of the study, according to these findings, the sample with the smallest blue shift accompanied by the largest absorbance intensity difference between the 325 nm valley and the SPR peak was chosen. Smaller molecular weight PVP proved to be more favorable for AgNP stabilization as smaller polymer chains in the same mass result in a larger number of single chains, which can spread out more evenly on the nanoparticle surfaces [28,29]. Gaining better PVP coverage by increasing the PVP concentration (from 2 to 10 mg/mL) proved to be ineffective as increasing the amount of PVP most likely pushed its concentration beyond a critical point, where polymer–polymer interactions became more prominent than polymer–nanoparticle interactions, leading to the reduced chemical stability of AgNPs in an environment containing large amounts of chlorine (AgNP samples mixed with 100 mM NaCl). The best results were produced with PVP of 40k average MW, applied in a concentration of 2 mg/mL. As this sample demonstrated better chemical uniformity as well, it was labeled AgNP@PVP and used in later experiments, and subsequent references to this abbreviation will always correspond to the sample stabilized by 2 mg/mL (13.3 mg/mg AgNP) 40k MW PVP.

### 2.3. Aggregation Behavior Assays

#### 2.3.1. The Effect of pH

**Figure 3:**
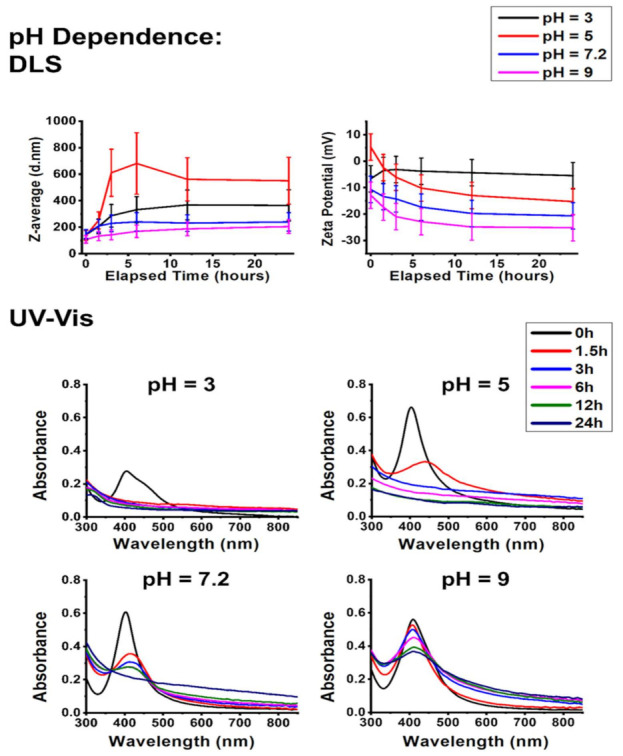
The effect of pH on the aggregation behavior of PVP-stabilized silver nanoparticles. Dynamic light scattering (DLS) results (top) are represented as average hydrodynamic diameter (Z-average) and zeta potential changes; UV–Vis absorbance spectra (bottom) of PVP-stabilized silver nanoparticles taken over 24 h under various pH values.

Figure 3 demonstrates the effects of pH on the colloidal stability of AgNP@PVP.

**Scheme 1:**
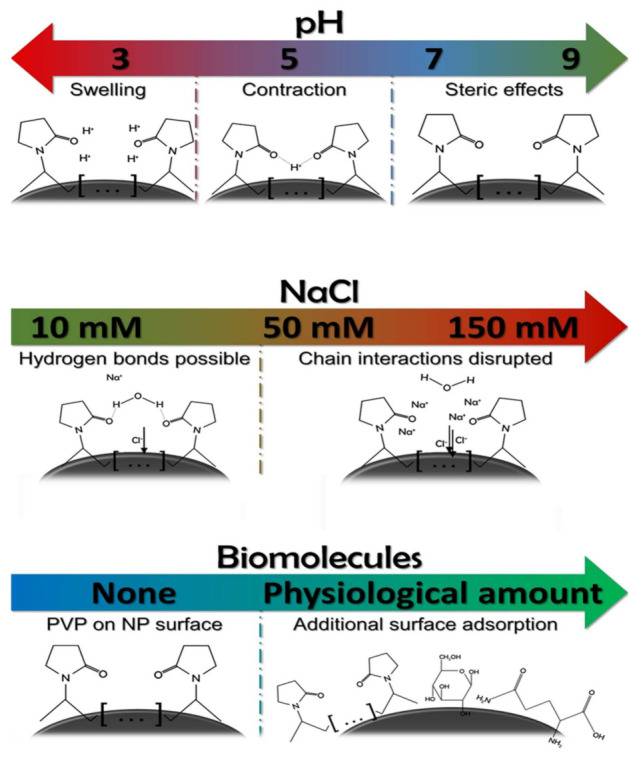
The effect of environmental conditions on the steric stabilization of silver nanoparticles protected by polyvinyl pyrrolidone.

According to the dynamic light scattering (DLS) and zeta potential results, the alkaline condition of pH 9 was the most favorable environment, closely followed by the neutral pH of 7.2. Under these circumstances, the smallest hydrodynamic diameters were detected, which did not increase substantially throughout the experiments. This finding is important since a significant rise in the hydrodynamic diameter would mean extensive particle aggregation [30,31]. Moreover, the lowest zeta potentials—a marker for high colloidal stability—were measured under pH 7.2 and pH 9 conditions as a result of the increasing OH^−^ concentration of the systems. However, a further decrease in the pH led to higher Z-average values under pH 5, which were higher than those obtained in a pH 3 environment. The proposed mechanism explaining these observations is surmised in Scheme 1.

At pH 5, the moderately increased H^+^ concentration allows the formation of “bridges” between pyrrolidone groups, contracting the AgNP cappings or possibly interconnecting adjacent AgNPs, resulting in an increased aggregation grade [32]. Nevertheless, further increase in the H^+^ concentration of the samples led to the swelling of PVP, in good agreement with the literature [33]. This swelling granted steric stabilization of the nanoparticles, and, therefore, lower average hydrodynamic diameters were detected at this pH.

In our previous publication, changes in the Z-average values were closely accompanied by alterations in the zeta potential, which was understandable, given the fact that the samples investigated there possessed stronger electrostatic characteristics [18]. However, AgNP@PVP is an overwhelmingly sterically stabilized system, and, as such, the interrelationship of hydrodynamic diameter and zeta potential and the changes within are not so straightforwardly linked. Even though the sample corresponding to pH 5 demonstrated the largest aggregates, the highest zeta values were detected at pH 3. The other three conditions all facilitated decreasing zeta potential tendencies. These observations can be explained by the gradual formation of AgCl precipitation on the surface of the nanoparticles, which is known to decrease the overall zeta potential, induced by the presence of the 10 mM NaCl background electrolyte concentration [30,34].

The DLS and zeta potential results are in close correlation with the UV–Vis spectra shown in Figure 3. Aggregation can be interpreted from the redshift and intensity decrease of the characteristic SPR absorbance peaks of AgNPs, while the formation of AgCl is once again demonstrated by the absorbance increase of the local minima before the SPR peaks (around 325 nm). Slight nanoparticle aggregation was observed in all samples, with increasing intensity towards acidic pH. Interestingly, a very steep SPR decline is observed on the spectra obtained at pH 3, although the spectral baselines did not increase. Thus, this feature is most likely due to the formation of compact aggregates rather than chemical decomposition through AgCl. This explains why the zeta potential of this system trended in the opposite direction compared to the rest of the pH-related experiments.

In summary, the colloidal stability of AgNPs stabilized by PVP remained largely unaffected on alkaline pH values, while mildly acidic pH caused notable aggregation due to the formation of H^+^ bridges among pyrrolidone groups. However, the chemical stability of the particles was not secured through steric stabilization, and the chemical decomposition of AgNPs via AgCl precipitation was noticeable.

#### 2.3.2. The Effect of NaCl

**Figure 4:**
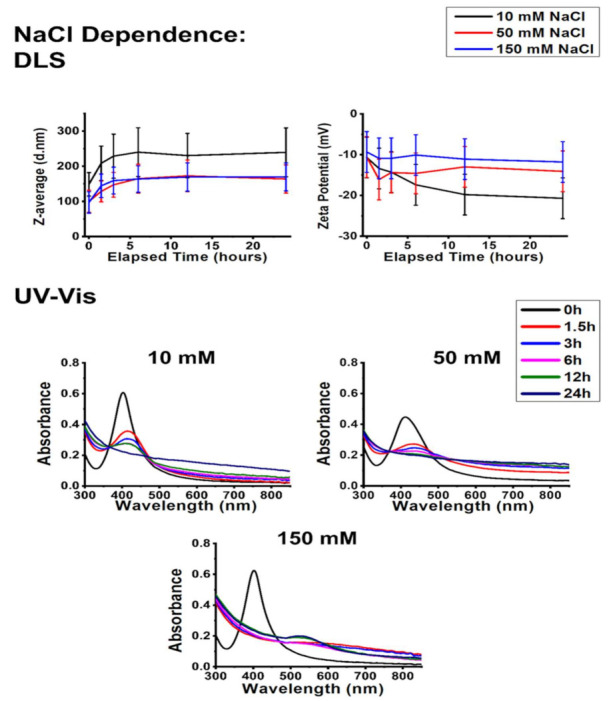
The effect of physiological NaCl concentrations on the stability of AgNP@PVP. Average hydrodynamic diameter (Z-average) and zeta potential changes (top panels) obtained by dynamic light scattering (DLS) and UV–Vis absorbance spectra (bottom panels) of polyvinyl-pyrrolidone-stabilized silver nanoparticles, recorded under various NaCl concentrations over a 24-h timeframe.

The impact of physiological NaCl concentrations on the stability of AgNP@PVP is represented in Figure 4. According to the results, increasing NaCl content somewhat decreased the average hydrodynamic diameter of the samples and caused an increase in their zeta potential. The UV–Vis results indicate AgCl generation, but this was not accompanied by zeta potential decrease, which was previously observed during the pH-related measurements. No SPR blueshift was detected (in fact, redshifts were visible on the absorbance spectra). Therefore, the decreased Z-average values cannot be explained solely by AgNP degradation despite that AgCl formation was substantial on physiological NaCl concentrations according to the UV–Vis graphs.

Upon reviewing the related literature, the interconnection of PVP molecules through PVP–water–PVP hydrogen bonds seems a plausible reason for these observations [35]. As the increasing NaCl concentration led to a decreased Z-average value while simultaneously increased the zeta potential of the nanoparticles, it can be hypothesized that the increasing Na^+^ ion presence at the slipping plane of the nanoparticles interfered with the interconnecting H-bonds among PVP chains, which resulted in a decrease of average aggregate size, illustrated in Scheme 1. This explains the observed optical changes within the samples since changes in the dielectric constant due to increasing electrolyte concentration can affect surface plasmon resonance [24].

#### 2.3.3. The Effect of Glucose and Glutamine

**Figure 5:**
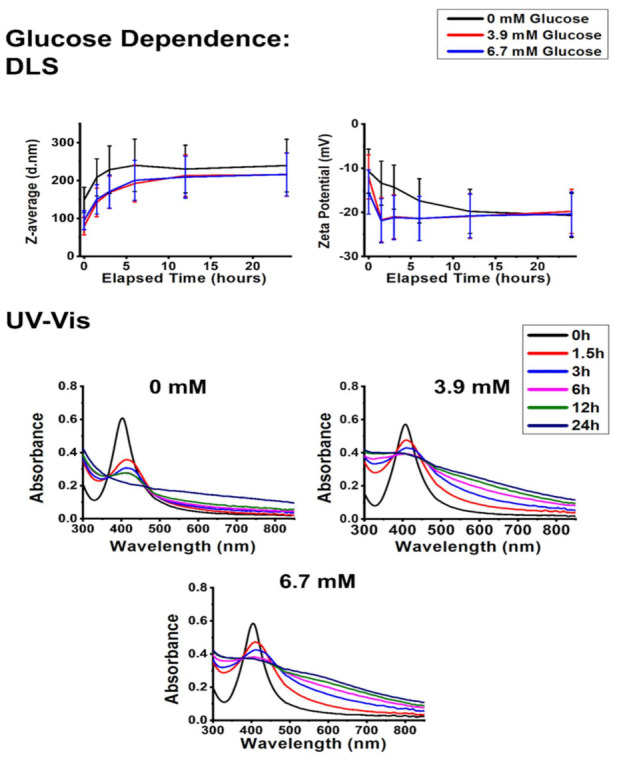
Dynamic light scattering (DLS) results (top), represented as average hydrodynamic diameter (Z-average) and zeta potential changes, and UV–Vis absorbance spectra (bottom) of polyvinyl-pyrrolidone-stabilized silver nanoparticles over 24 h under physiological glucose concentrations.

**Figure 6:**
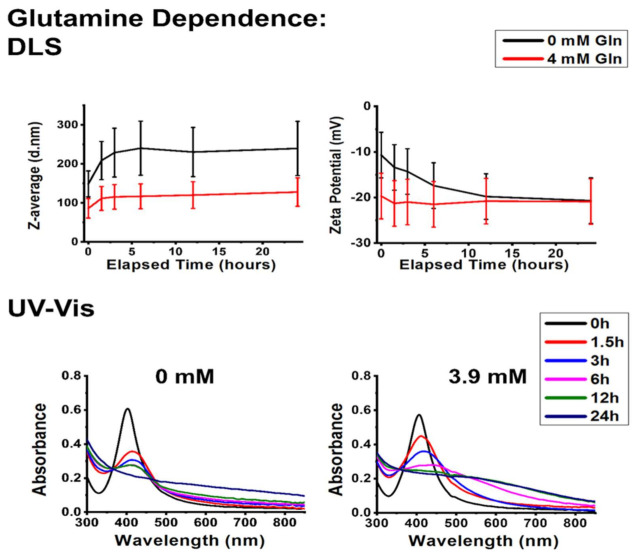
The presence of glutamine affects the stability of polyvinyl-pyrrolidone-stabilized silver nanoparticles. The average hydrodynamic diameter (Z-average) and zeta potential values were assessed by dynamic light scattering (DLS) (top) and UV–Vis absorbance spectra (bottom) of AgNP@PVP, recorded over 24 h in the presence of glutamine.

According to Figure 5 and Figure 6, the addition of small biomolecules to the colloids induced similar changes in the aggregation behavior of silver nanoparticles, regardless of the chemical composition or the concentration of the given biomolecule. Based on the DLS measurements taken at the early time points (between 1.5 and 6 h), the presence of glucose in the physiological concentration and the application of glutamine in a concentration typically used in in vitro cell culture systems both decreased aggregate diameters and zeta potentials, ultimately converging towards the reference experiments (where the measurement was carried out on pH 7.2 without interfering agents). The UV–Vis spectra of the colloids placed under these circumstances imply that the zeta potential results cannot be simply explained by AgCl formation as the characteristic AgNP SPR peaks were detectable (albeit redshifted) even after 24 h. These results suggest that the investigated biomolecules get adsorbed on the NP surfaces, which feature moderately improved chemical and colloidal stability [36,37]. This surface adsorption (Scheme 1) is commonly referred to as biomolecular corona formation in more complex systems, and it can affect the optical and colloidal properties of AgNPs [25,38,39].

#### 2.3.4. The Effect of Cell Culture Medium Components

In this section, the effect of two different cell culture components, Dulbecco’s modified Eagle’s medium (DMEM) and fetal bovine serum (FBS), was investigated on nanoparticle aggregation. From a chemical standpoint, DMEM can be considered a complex salt solution, and FBS a mixture of various biomolecules, overwhelmingly proteins [40]. Recognizing these solutions as chemically similar analogs for an environment of differing NaCl concentrations, tested previously, and another environment containing biomolecules such as glucose/glutamine, also examined in the preceding experiments, the data gathered here can be easily compared with the results described above in the manuscript.

**Figure 7:**
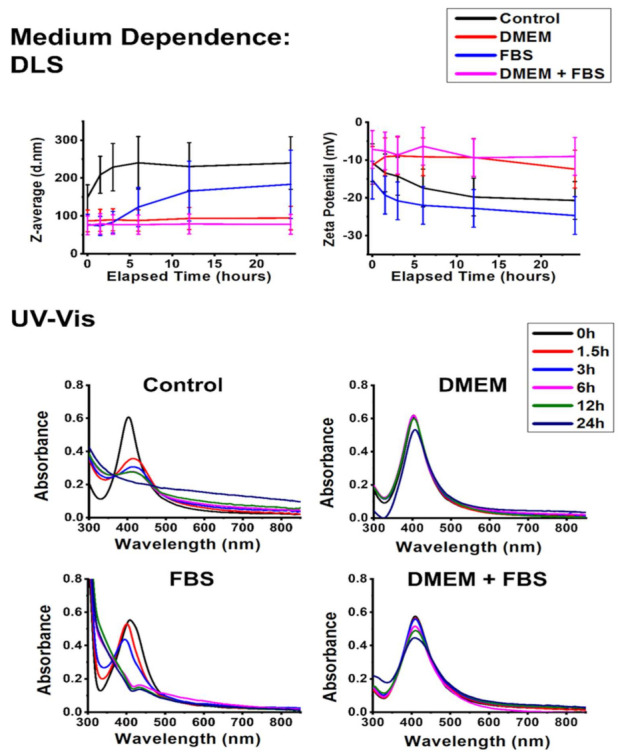
Aggregation behavior of silver nanoparticles characterized by dynamic light scattering (DLS) measurements (top) (average hydrodynamic diameter (Z-average) and zeta potential) and UV–Vis absorbance spectra (bottom) in the presence of cell culture components DMEM and FBS over a 24 h interval.

Compared to the control experiment, the addition of DMEM slightly decreased the average hydrodynamic diameter and zeta potential and simultaneously stabilized the SPR absorbance of the particles. As a result of those experiments, where we assessed the effects of NaCl on particle aggregation, the accumulation of Na^+^ ions around the slipping plane was hypothesized as the guiding feature behind the size decrease of AgNP@PVP aggregates. In those experiments, this phenomenon was accompanied by AgCl formation, indicated by ζ-potential decrease and UV–Vis spectral changes. Here, in the presence of DMEM, we found a similar trend (Figure 7); however, silver chloride precipitation was not detected based on the UV–Vis spectra. The observation can be justified by inspecting the composition of DMEM. The same ionic strength is achieved by the addition of DMEM as NaCl (see in Section 2.3.2), only with a lower Cl^−^ content; thus, the chemical stability of AgNPs was not so severely compromised under these conditions as within the milieu of the NaCl solution.

FBS induced analogous changes in the stability of AgNP@PVP just as glucose or glutamine did, with a moderately decreased Z-average and increased zeta potential. However, the presence of FBS induced a rather unique effect on the SPR of AgNPs, revealing interesting details of the mechanisms associated with nanosilver degradation due to NaCl. According to the literature, the observed changes of SPR characteristics in the UV–Vis spectra can be attributed to the formation of clusters consisting of silver or silver chloride [41,42]. These clusters were most likely formed in the previous experiments as well but were immediately converted to bulk AgCl precipitation. The presence of FBS, however, prevented this process by creating a protective corona around the clusters. At the same time, it also displays how the chemical degradation of AgNPs due to NaCl involves a certain degree of particle fragmentation. These observations highlight that biomolecular coronas are not simply accessories for immune reactions, which is their most prominently discussed feature in the literature, but manifest an even more valuable characteristic as colloidal stabilizers of nanoparticles under biorelevant conditions [18,39,43,44].

As DMEM and FBS both improved the overall chemical and colloidal stability of AgNP@PVP particles separately, their combination was expected to exert a positive effect. Indeed, the DMEM+FBS experiment provided the smallest detected aggregate sizes within the entire work. The ζ-potential of the system increased similarly to what was observed in the presence of only FBS, indicating considerable surface adsorption of various compounds. In contrast, the UV–Vis spectra of AgNP@PVP did not change substantially throughout the experiment despite a slight broadening, redshift, and intensity decline, which complemented the corona formation suggested by the zeta potential results.

The information gathered from the experiments revealed that during the biological application of AgNP@PVP, chemical stability rather than colloidal stability is the main point of contention; the latter proved to be the biggest weakness of nanosilver samples prepared with electrostatic stabilization, e.g., citrate groups [18].

### 2.4. Biological Activity

#### 2.4.1. Toxicity on Human Cells

In order to evaluate how cytotoxic propensity can be influenced by the different aggregation states of AgNPs, we decided to use a two-step MTT cell viability assay-based approach, utilizing human cervical cancer (HeLa) cells as well as immortalized human keratinocyte (HaCaT) cells.

For these analyses, in the first step, cell viability was measured after exposing both cell lines to AgNP@PVP in different concentrations to obtain the corresponding IC_50_ values. The IC_50_ values on HeLa cells were found to be 15.22 ppm, whereas IC_50_ values on HaCaT cells were 2.74 ppm. Then, in the second step, the changes in AgNP cytotoxicity as the function of particle aggregation were explored in a time-dependent manner. For this, different silver nanoparticle aggregation states were formulated by adding 150 mM NaCl to silver samples for 0, 1.5, 3, 6, 12, and 24 h prior to the beginning of the toxicity assays. We selected this approach based on our preliminary results and previous contributions, which indicated that among the tested experimental conditions, the electrolyte concentration had the greatest impact on nanoparticle stability [18,19]. AgNP samples of differing aggregation degrees (NaCl-treated AgNP samples) were created at nanoparticle concentrations corresponding to the respective cell line-specific IC_50_; then, the cells were incubated for 24 h with these AgNP samples, followed by MTT experiments. This way, the aggregation-dependent toxicity of AgNP@PVP samples could be measured.

**Figure 8:**
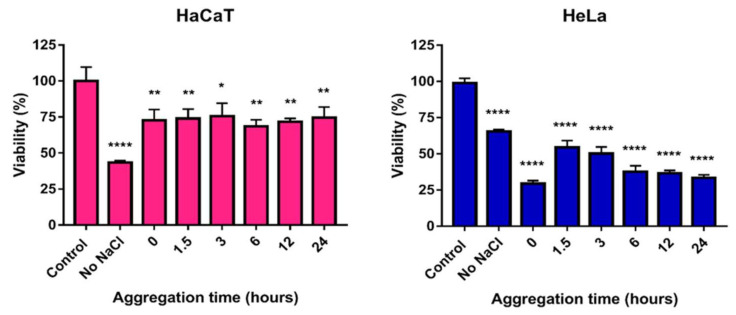
The effect of nanoparticle aggregation on the cytotoxicity exhibited on immortalized human keratinocyte (HaCaT) cells and human cervical cancer (HeLa) cells. Increasing aggregation states of PVP-stabilized silver nanoparticles were created by incubating the particles with 150 mM NaCl for different time periods (0, 1.5, 3, 6, 12, and 24 h) prior to cell treatments. The statistical significance of the experiments was calculated through unpaired *t*-tests and are marked with * (*p* ≤ 0.05), ** (*p* ≤ 0.01), and **** (*p* ≤ 0.0001).

The MTT viability assays performed on HeLa cervical cancer cells and the HaCaT keratinocyte cell line revealed that the toxicity of PVP-stabilized nanoparticles remained largely unchanged with increasing aggregation grades (Figure 8). These results implied that no aggregates were formed and all investigated AgNPs were stable and preserved their colloidal stability and toxicity for the entire aggregation timeframe. Despite the observed colloidal stability, by this point, we knew that the chemical stability of the particles was, in fact, compromised (Figure 2 and Figure 4) since these particles were prone to decomposition upon surface contact with Cl^−^ ions through AgCl formation. Therefore, the results reflected the combined effect of AgNP@PVP and the generated AgCl. Consequently, the IC_50_ values of AgCl on both cell lines were determined as well, which were comparable with the IC_50_ values of AgNP@PVP (IC_50_ values of AgCl: 10.37 ppm on HeLa cells; 2.31 ppm on HaCaT cells). These results are in good agreement with the fact that toxicity was maintained at the same level throughout the experimental time frame.

#### 2.4.2. Antimicrobial Activity

In order to provide the most reliable results, the changes in antibacterial and antifungal activities of AgNP@PVP samples in differing aggregation grades were also examined.

For these, a similar procedure to the cytotoxicity studies on human cells was used. The minimal inhibitory concentrations (MICs) were defined first and then used throughout the microbiology experiments. Then, the antimicrobial effect of the NaCl-treated silver samples (where the aggregation grade was modulated by the addition of NaCl) was tested against *Cryptococcus neoformans*, *Bacillus megaterium,* and *Escherichia coli* strains. The selection of the pathogens was based on the idea of having a representative strain of Gram (+) and Gram (-) bacteria as well as of eukaryotic microorganisms.

**Figure 9:**
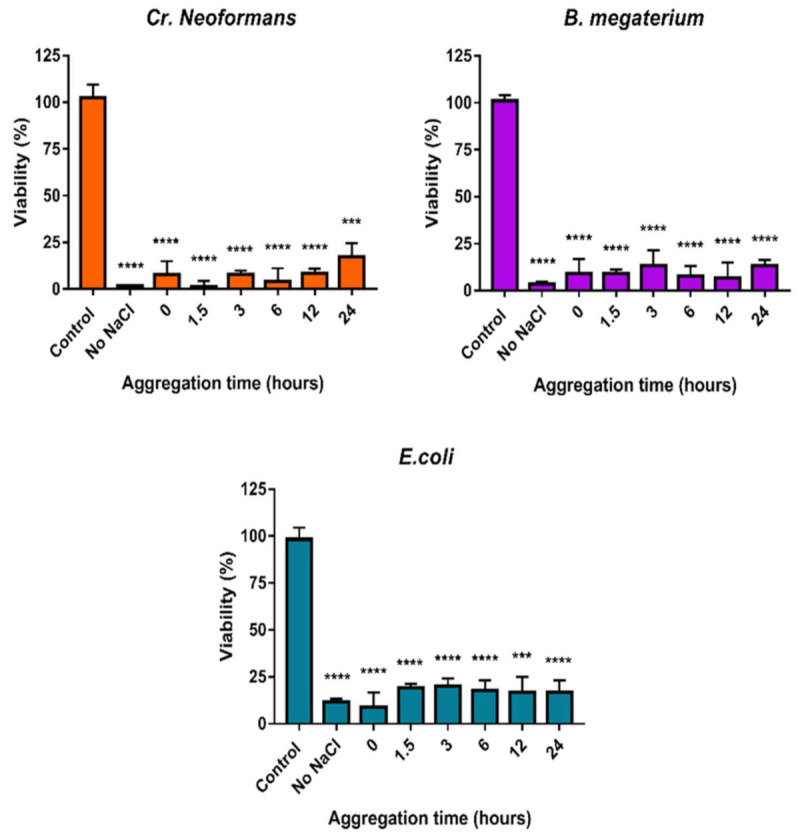
The effect of nanoparticle aggregation on the antimicrobial activity against *Cr. neoformans*, *B. megaterium*, and *E. coli*. Increasing aggregation degree was achieved by incubating the particles with 150 mM NaCl for different time periods (0, 1.5, 3, 6, 12, and 24 h) prior to treatments. The statistical significance of the experiments was calculated through unpaired *t*-tests and are marked with *** (*p* ≤ 0.001), and **** (*p* ≤ 0.0001).

The minimal inhibitory concentration of AgNP@PVP was 3.90 ppm for *B. megaterium* and *E. coli* and 1.95 ppm for *Cr. neoformans*. We found that the viability of all three strains remained unaffected; the antimicrobial efficiency of the nanoparticles remained the same even under growing nanoparticle aggregation grades (Figure 9). This was in accordance with the cytotoxicity results. In all cases, microbial cell viability was the lowest when cells were exposed to AgNPs that were not previously treated with NaCl. In the case of samples where NaCl was supplemented to induce particle aggregation, the toxicity was not affected by the loss of particle stability; only a small difference in the extent of the toxic effect could be noticed. *Cr. neoformans* was the most sensitive, and *E. coli* was the most resistant strain against AgNP@PVP. In line with the human toxicity data, we concluded that AgNP@PVP samples could retain a significant degree of inhibitory activity on the tested microbes for the entire experimental timespan (~above 80% effectiveness for all strains).

It is not sufficiently emphasized, but nanoparticle stability is an important physicochemical feature that must be seriously considered when nanoscale materials are designed and produced since aggregation could be a huge barrier in efficient nanoparticle application. It is known that the various materials used for stabilization have a defining role in determining and fine-tuning the biological activities of the obtained nanoparticles, such as toxicity, anticancer features, or antimicrobial activity, all of which can be seriously compromised by particle aggregation [45]. In our previous research, we found that the electrostatic repulsion provided by the most commonly used stabilization method, i.e., citrate capping, results in nanoparticles that are highly susceptible towards aggregation under biorelevant conditions, which can lead to the complete loss of their toxicity in specific circumstances. The results suggest that the loss of toxicity can be counteracted by either increasing the primary nanoparticle size or the introduction of additional repulsive forces among particles or through stronger (electrosteric) capping or biomolecular corona formation [18,19,46,47]. The use of PVP as a biocompatible and robust steric stabilizer was a suitable continuation of this research, as PVP already has numerous relevant uses, from the food industry to the pharmaceutical industry and drug delivery [32,33]. Our comprehensive chemical and biological data verify that PVP-stabilized silver nanoparticles are quite resistant against aggregation under biorelevant conditions and demonstrate the highest time-dependent toxicity we have reported to date. However, the chemical stability of the particles was weaker than what we observed within the systems of our previous contributions, where electrostatic repulsive interactions were also utilized. Interestingly, the degradation of the particles via AgCl precipitation did not substantially affect the toxicity of the particles, indicating that colloidal stability might be more important than chemical stability regarding the longevity of silver nanoparticle toxicity. However, it should be considered that biomedical (especially therapeutic) applications generally require well-defined, unchanging, long-term chemical characteristics and biological effects, which, according to our results, might not be plausible through PVP capping alone. On the other hand, certain environmental applications (for instance, wastewater treatment) could potentially benefit from a nanoparticulate system that can be administered in a way where the initial nano-size properties can be effectively utilized while the separation of the nanomaterial can be realized through the removal of the water-insoluble precipitation that is generated over time.

## 3. Materials and Methods

### 3.1. Nanoparticle Synthesis

All chemicals were purchased from Merck KGaA (Darmstadt, Germany). PVP-stabilized silver nanoparticles (AgNP@PVP) were prepared by chemical reduction based on the approach proposed by Wan et al. with slight adjustments, the same procedure that we used in our previous contributions [18,48,49]. While the original method is based on the chemical reduction of silver nitrate (AgNO_3_) by sodium borohydride (NaBH_4_) with sodium citrate capping, here we used PVP in two different (40k and 55k) average molecular weights as stabilizing agents. Firstly, 0.17 g PVP (40k or 50k) was dispersed in 96 mL of ion-exchanged water and was heated to 70 °C in a beaker. Then, 2 mL 1% *w*/*v* AgNO_3_ solution was administered to the polymer solution, followed by the dropwise addition of 2 mL 0.1% *w*/*v* freshly prepared sodium borohydride. The resulting AgNP samples were kept at 70 °C under vigorous stirring for 1 h and, afterward, left to cool to room temperature. Any remaining contaminants were removed by dialysis using a cellulose membrane. Ultimately, the volumes of the samples were set to 85 mL; thus, each suspension was 150 ppm regarding AgNPs and 2 mg/mL (or 13.3 mg/mg AgNP) for PVP, respectively.

### 3.2. Nanoparticle Characterization

The morphological characteristics of the nanoparticles were assessed by transmission electron microscopy (TEM) with an FEI Tecnai G^2^ 20 X-Twin instrument using 200 kV accelerating voltage (FEI Corporate Headquarters, Hillsboro, OR, USA). The size distribution of the samples was demonstrated through histograms assessed by evaluating 15 representative images. The crystallinity and, thus, the chemical composition of the particles was verified through electron diffraction (ED).

### 3.3. Selection of the Proper AgNP@PVP System Based on Chemical Stability

Preliminary experiments were performed on an Ocean Optics 355 DH-2000-BAL UV–Vis spectrophotometer (Halma PLC, Largo, FL, USA) to investigate the chemical stability of AgNPs in the abundance of chloride ions by observing changes in the surface plasmon resonance (SPR) of the nanosilver samples [50]. To identify the most chemically resistant AgNP@PVP system produced via the synthesis method, both AgNP samples (stabilized by 40k and 55k molecular weight PVP, respectively) were examined by UV–Vis spectroscopy. The PVP concentrations of the as-prepared samples were adjusted with the appropriate molecular weight polymer to 2, 4, 8, and 10 mg/mL (or 13.3, 26.6, 53.3, and 66.6 mg/mg AgNP, respectively), and their initial SPR spectra were collected after a 30 s long sonication. Then, the samples’ NaCl concentrations were set to 100 mM, and their UV–Vis absorbance was once again measured after 20 h. The best sample regarding the molecular weight and concentration of PVP was identified and used for the subsequent colloidal stability experiments.

### 3.4. Aggregation Behavior Assays

We have previously demonstrated that biologically relevant conditions may affect the stability and the behavior of citrate-capped and green-tea-extract-stabilized silver nanoparticles; furthermore, in a following contribution, we highlighted the importance of primer particle size as a parameter with profound importance on colloidal stability and, hence, toxicity using a novel experimental setup of our own [18,19]. As the applied methodological approach proved to be adequate and highly competent to reliably characterize these features, we performed the same methodology here to describe the aggregation propensity of the selected AgNP@PVP sample. The effect of pH, sodium chloride, glucose, and glutamine, as well as Dulbecco’s modified Eagle medium (DMEM, Sigma-Aldrich, Saint Louis, MO, USA) and fetal bovine serum (FBS, Sigma-Aldrich, Saint Louis, MO, USA) (both in water and DMEM) were investigated at biologically and environmentally important values and concentrations, according to Table 1 [51,52,53,54,55]. Each experiment lasted 24 h, with measurements performed at the 0, 1.5, 3, 6, 12, and 24 h checkpoints. At the checkpoints, the characteristic UV–Vis spectra of the samples were captured; furthermore, the Z-average (using dynamic light scattering) and ζ-potential of the samples were also investigated on a Malvern Zetasizer Nano ZS instrument (Malvern Instruments, Malvern, UK). In all experiments, the concentration of AgNPs was set to 22 ppm. This nanoparticle concentration was selected because this concentration corresponded to ~0.8 absorbance, allowing the low-noise and sensitive detection of spectral changes; furthermore, this way, all considered biorelevant agents were in concentrations of higher orders of magnitude compared to that of nanoparticles, adequately simulating the lifelike circumstances. Unless otherwise stated, all measurements were performed at pH 7.2 and 37 °C, with a 10 mM NaCl background salt concentration to negate long-distance particle interactions; furthermore, the pH of the samples was always checked before measurements and corrected when necessary [56].

### 3.5. Cytotoxicity Assays

Human cervical carcinoma (HeLa) cells and immortalized human keratinocyte (HaCaT) cells were purchased from American Type Culture Collection (ATCC, Manassas, VA, USA). Cells were routinely maintained in DMEM containing 4.5 g/L glucose (Sigma-Aldrich, Saint Louis, MO, USA), supplemented with 10% FBS, 2 mM L-glutamine, 0.01% streptomycin, and 0.005% penicillin (all components were obtained from Sigma-Aldrich, Saint Louis, MO, USA). Cells were cultured under standard conditions in a 37 °C incubator at 5% CO_2_ in 95% humidity.

Similar to our previous experiments, a well-defined system was employed to measure the changes in AgNP cytotoxicity affected by particle aggregation in a time-dependent manner, as proposed by our previous contributions [18,19]. A two-step approach based on MTT assays was carried out, where, first, the IC_50_ values of the PVP-stabilized silver sol were determined as a reference for the toxicity of non-aggregated particles for both the cancerous and healthy human cell lines, and the effect of aggregation was demonstrated on these concentrations in a subsequent round of MTT assays. For this, both types of cells were seeded into 96-well plates in a 10^4^ cells/well density and treated on the following day with silver nanoparticles in increasing concentrations. After 24 h treatments, cells were washed with PBS and incubated with a culture medium containing 0.5 mg/mL MTT reagent (Sigma-Aldrich, Saint Louis, MO, USA) for 1 h at 37 °C. The resulting formazan crystals were dissolved in DMSO (Sigma-Aldrich, Saint Louis, MO, USA), and the absorbance of the samples was measured at 570 nm using a SPECTROstar Nano plate reader (BioTech-Hungary, Budapest, Hungary). Absorption values of the untreated control samples were considered 100% viability. The experiments were carried out three times using four independent biological replicates. IC_50_ was calculated using the dose–response curve based on the viability results.

Thereafter, different silver nanoparticle aggregation states were formulated by incubating the particles (at the IC_50_ concentration) with 150 mM NaCl for different time periods (0, 1.5, 3, 6, 12, and 24 h) prior to cell treatments. Subsequently, the MTT assay was performed, as described above, to explore the changes in cell viability caused by particle aggregation.

### 3.6. Assessment of the Antimicrobial Activity

To evaluate the changes in antimicrobial activities caused by particle aggregation, first, a microdilution method was employed to determine the minimum inhibitory concentration (MIC) of the nanosilver sample as a reference for the toxicity of non-aggregated particles. The MIC was defined against microbial strains that were the subjects of our previous studies [18,19]. The initial cell concentration of *Cryptococcus neoformans* IFM 5844 (IFM; Research Center for Pathogenic Fungi and Microbial Toxicoses, Chiba University), *Bacillus megaterium* SZMC 6031, and *Escherichia coli* SZMC 0582 (SZMC: Szeged Microbiology Collection) strains in RPMI 1640 medium (Sigma-Aldrich, Saint Louis, MO, USA) was 10^5^ cells/mL, respectively. Two-fold dilution series of nanoparticles were made in the 0–75 ppm concentration range. From each cell suspension, 50 μL aliquots were loaded into the wells of a 96-well microplate, and 50 μL of individual AgNP dilutions were added to the cells. Suspensions supplemented with 50 μL RPMI 1640 were used as growth control. Then, microplates were incubated at 30 °C for 48 h, and the optical density of the cultures was detected at 620 nm using a SPECTROstar Nano plate reader (BMG LabTech, Offenburg, Germany). The experiments were carried out three times in triplicates.

The antimicrobial activity of the aggregated nanoparticles was tested on the above-mentioned strains with the same approach described in the previous paper [18]. Briefly, suspensions of 10^5^ cells/mL were prepared in RPMI 1640 medium. Then, 50 μL aliquots from *Cr. neoformans*, *B. megaterium*, and *E. coli* cell suspensions were loaded into the wells of a 96-well microplate, and, this time, 50 μL of the individual aggregated nanoparticle samples were added to the cells. The different silver nanoparticle aggregation states were created by incubating the particles (at the MIC concentration) with 150 mM NaCl for different time periods (0, 1.5, 3, 6, 12, and 24 h) before cell treatments. Suspensions supplemented with 50 μL RPMI 1640 were used as growth control, while suspensions with non-aggregated nanoparticles were used as controls of toxicity. The light absorbance of the samples was measured at 620 nm using a SPECTROstar Nano plate reader (BMG LabTech, Offenburg, Germany). The experiments were carried out three times in four replicates.

### 3.7. Statistical Analysis

All the experiments were carried out three times in four replicates. The final results of the cytotoxicity and antimicrobial assays were assessed using GraphPad Prism 7 (GraphPad Software, San Diego, CA, USA); the statistical significance of the experiments was calculated through unpaired *t*-tests, and their levels are marked as * (*p* ≤ 0.05), ** (*p* ≤ 0.01), *** (*p* ≤ 0.001), and **** (*p* ≤ 0.0001).

## 4. Conclusions

Silver nanoparticles are among the most biologically significant nano-sized materials due to their exceptional antimicrobial, anti-inflammatory, anticancer, and antiangiogenic properties. However, due to nanoparticle aggregation—a phenomenon often occurring in biological conditions—these advantageous characteristics are easily perturbed. In this work, the effectiveness of PVP as a silver nanoparticle-capping agent was assessed against biologically relevant materials and conditions. The first objective of our research was the synthesis and characterization of polymer-capped silver nanoparticle samples stabilized by 40k or 55k MW PVP, aiming to find a sample with high chemical uniformity and stability. In the following steps, through the subsequent aggregation behavior experiments, the sample possessing the best characteristics (AgNP@PVP, stabilized by 40k MW PVP in 13.3 mg/mg AgNP relative concentration) demonstrated robust colloidal stability against most of the investigated conditions apart from mildly acidic pH, where the crosslinking of polymer chains became possible through H^+^ bridges. Despite the outstanding colloidal stability of the sample, the chemical degradation of AgNPs through AgCl precipitation was detected in the presence of elevated Cl^−^ concentrations. While chemical degradation could be observed with as low a NaCl concentration as 10 mM, biomolecular corona formation demonstrated a protective effect against precipitation via the surface adsorption of biomolecules on nanoparticle surfaces. In fact, corona formation revealed the presence of silver clusters in the precipitating samples, indicating that the chemical degradation of AgNPs under biorelevant conditions might involve nanoparticle fragmentation. Throughout the in vitro studies, AgNP@PVP demonstrated excellent and long-lasting toxicity against cancerous and non-cancerous human cell lines and microbial strains alike. In contrast with our previous contributions, it can be assessed that colloidal stability is more important than chemical stability for the longevity of silver nanoparticle toxicity, although when facing real biomedical challenges, such as in vivo therapeutic applications, extra attention must be paid to exclude the possibility of adverse reactions due to precipitation.

## Figures and Tables

**Table 1:** List of applied biorelevant conditions; (^†^) represents the reference point for the measurements. All samples were measured at 37 °C, pH~7.2, with a 10 mM NaCl electrolyte background unless otherwise stated.

Condition	Value(s)
pH	3; 5; 7.2 ^†^; 9
NaCl concentration (mM)	10; 50; 150
Glucose concentration (mM)	3.9; 6.7
Glutamine concentration (mM)	4
DMEM (*v*/*v*%)	45
FBS (*v*/*v*%, both in water and DMEM mixture)	5

## Data Availability

The data presented in this study are available on request from the corresponding author.
